# Cost-effectiveness of a stepped-care intervention to prevent major depression in patients with type 2 diabetes mellitus and/or coronary heart disease and subthreshold depression: design of a cluster-randomized controlled trial

**DOI:** 10.1186/1471-244X-13-128

**Published:** 2013-05-07

**Authors:** Susan EM van Dijk, Alide D Pols, Marcel C Adriaanse, Judith E Bosmans, Petra JM Elders, Harm WJ van Marwijk, Maurits W van Tulder

**Affiliations:** 1 Department of Health Sciences and the EMGO institute for Health and Care research, Faculty of Earth and Life Sciences, VU University Amsterdam, De Boelelaan 1085, Amsterdam, HV, 1081, The Netherlands; 2 Department of General Practice and the EMGO institute for Health and Care research, VU University Medical Centre, Amsterdam, The Netherlands

**Keywords:** Subthreshold depression, Depression prevention, Cost-effectiveness, Type 2 diabetes mellitus, Coronary heart disease, Stepped-care, Primary care, Nurse led treatment

## Abstract

**Background:**

Co-morbid major depression is a significant problem among patients with type 2 diabetes mellitus and/or coronary heart disease and this negatively impacts quality of life. Subthreshold depression is the most important risk factor for the development of major depression. Given the highly significant association between depression and adverse health outcomes and the limited capacity for depression treatment in primary care, there is an urgent need for interventions that successfully prevent the transition from subthreshold depression into a major depressive disorder. Nurse led stepped-care is a promising way to accomplish this. The aim of this study is to evaluate the cost-effectiveness of a nurse-led indicated stepped-care program to prevent major depression among patients with type 2 diabetes mellitus and/or coronary heart disease in primary care who also have subthreshold depressive symptoms.

**Methods/design:**

An economic evaluation will be conducted alongside a cluster-randomized controlled trial in approximately thirty general practices in the Netherlands. Randomization takes place at the level of participating practice nurses. We aim to include 236 participants who will either receive a nurse-led indicated stepped-care program for depressive symptoms or care as usual. The stepped-care program consists of four sequential but flexible treatment steps: 1) watchful waiting, 2) guided self-help treatment, 3) problem solving treatment and 4) referral to the general practitioner. The primary clinical outcome measure is the cumulative incidence of major depressive disorder as measured with the Mini International Neuropsychiatric Interview. Secondary outcomes include severity of depressive symptoms, quality of life, anxiety and physical outcomes. Costs will be measured from a societal perspective and include health care utilization, medication and lost productivity costs. Measurements will be performed at baseline and 3, 6, 9 and 12 months.

**Discussion:**

The intervention being investigated is expected to prevent new cases of depression among people with type 2 diabetes mellitus and/or coronary heart disease and subthreshold depression, with subsequent beneficial effects on quality of life, clinical outcomes and health care costs. When proven cost-effective, the program provides a viable treatment option in the Dutch primary care system.

**Trial registration:**

Dutch Trial Register NTR3715

## Background

Subthreshold depression, the presence of symptoms of depression without fulfilling the criteria for major depression, is the strongest predictor for the onset of major depression [1,2]. Currently, major depression is a substantial health problem throughout the industrialised world [3]. The 12 month prevalence of major depression ranges between 3 and 9% in high-income countries [4,5]. Among patients with type 2 diabetes mellitus (DM2) and/or coronary heart disease (CHD) estimates of the 12 month prevalence of major depression usually range from 10% to 20% [6-8]. Even more patients with DM2 and/or CHD experience subthreshold depression, i.e. 25%-40% [9,10]. More than 40% of diabetic patients with subthreshold depression will develop major depression within two years [9]. Similar estimates are found for patients diagnosed with CHD [11].

Depression has been shown to adversely affect self-care and medication adherence related to DM2 and CHD [12,13], to negatively impact quality of life [14,15] and to be associated with poor health outcomes and an increased risk of mortality [16,17]. Moreover, DM2 and CHD patients with depression use healthcare services more often than their non-depressed counterparts, which is associated with a substantial increase in health care related costs. This effect cannot be explained by an increase in mental health care costs alone and remains present after adjusting for co-morbid medical conditions [18-21]. In addition, depression is associated with increased work absenteeism, which raises the total societal costs of depression even more [22].

Unfortunately, even when optimal treatment is given to all patients with major depression, only about 27 percent of the total disease burden can be averted [23]. Therefore, prevention of the onset of major depression among high risk patients may be a promising solution to reduce the burden for both patients and society substantially [24].

Meta analyses show that using preventive interventions, a reduction of about 25% in the incidence of major depression can be obtained [2,25]. Especially promising are preventive interventions that are offered in a stepped-care format [25]. The aim of stepped-care interventions is to maximize the effectiveness of an intervention while making best use of available resources by offering the least intensive treatment necessary and by tailoring the treatment to the patient’s preferences. By using such a format, it is possible to reduce the relative risk of the onset of depression by as much as 50% [25-27].

There is also evidence that stepped-care interventions can be used to treat existing major depression among patients with DM2 in primary care [28]. This stepped-care depression treatment was shown to be cost-effective in comparison with usual care as well. The additional costs associated with the implementation of the stepped-care program did not result in higher total health care costs over respectively a 2- and 5- year period [29,30]. This effect remained after adjusting for co-morbid medical conditions. A more recent study showed that a stepped-care program is effective in improving disease control in chronically ill patients with existing major depression [31]. All these findings combined suggest that stepped-care is a promising method to not only treat major depression in patients with DM2 and/or CHD, but also to prevent the onset of this disorder in these patient groups. However, to the best of our knowledge, there are no studies evaluating the cost-effectiveness of a stepped-care program to prevent depression among DM2 and CHD patients.

Therefore, this study aims to evaluate the cost-effectiveness of a nurse-led indicated stepped-care program to prevent depression among primary care patients with type 2 diabetes mellitus and/or coronary heart disease and subthreshold depression in comparison with usual care.

## Methods/design

### Design

An economic evaluation from a societal perspective will be performed alongside a multi-center, cluster randomized controlled trial with a one year follow up.

### Ethical approval

The study protocol was approved by the Ethics Committee of the VU University Medical Centre (NL39261.029.12, registration number 2012/223) and will be conducted according to the principles of the Declaration of Helsinki (version 2008) and the Dutch Medical Research Involving Human Subjects Act (WMO).

### Setting

The study will be carried out in approximately thirty general practices in the Netherlands. To select these practices, we will make use of the Academic Network of General Practitioners (GPs) of the Department of General practice and of the VU medical centre.

### Randomization

Randomization will be done at the level of the participating practice nurses to avoid contamination between the treatment groups and will be performed by a statistician blinded to characteristics of the general practices using a computer generated list of random numbers. Before patients are recruited, participating practices will be randomly allocated to serve as intervention practices where the stepped-care treatment will be implemented, and control practices where care as usual will be given. Patients will be allocated to either one of the treatment conditions, based on the general practice where they are registered. Blinding of patients, GPs and practice nurses is not possible due to the nature of the intervention.

### Participants

Patients are eligible for this study if they are 18 years or older, are treated for DM2 and/or CHD in primary care, and have subthreshold depressive symptoms (a score of 6 or more on the Patient Health Questionnaire-9, or PHQ-9 [32]) without fulfilling the criteria for major depression according to the Diagnostic and Statistical Manual of Mental Disorders (DSM-IV), as measured with the Mini International Neuropsychiatric Interview (MINI) [33].

Patients are excluded from participation in the study when they have a major depressive disorder; cognitive impairment or dementia; a psychotic or terminal illness; a history of (a) suicide attempt(s); or insufficient Dutch language skills, visual impairments or illiteracy. Also, patients cannot participate when they are pregnant, taking antidepressant medication, or when they have lost a significant other in the past 6 months.

### Recruitment

**Figure 1:**
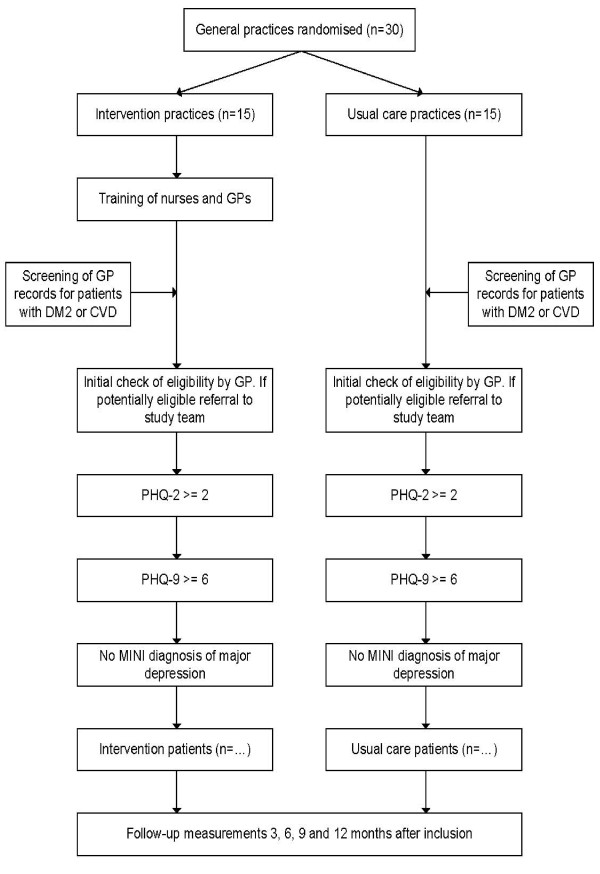
Overview of the study design and patient flow.

To recruit eligible patients, in all participating general practices an initial list of adult patients with DM2 and/or CHD is composed, based on diagnoses classified using the ICPC (International Classification of Primary Care) in the medical electronic information system; see Additional file 1 for details. This list is given to the general practitioner, who checks the preset inclusion criteria and excludes all patients that fulfill our preset exclusion criteria, based on the medical file and experiences with the patient. All remaining eligible patients will receive a letter on behalf of the general practitioner, in which all (at that point) necessary information regarding the study, as well as an invitation to participate is included. When patients consider to participate, they are asked to fill out a two question screening form (Patient Health Questonnaire-2, or PHQ-2 [34]) that will be provided with the information letter and send it back using a prestamped envelope. All patients with a PHQ-2 score of 2 or more [34,35] will receive additional information from the research team. Based on this information informed consent is obtained for a telephone interview. During this interview the Dutch version of the PHQ-9 [36] and the MINI [37] will be administered. Patients scoring 6 points or more on the PHQ-9, but not having a major depression according to the MINI are eligible for the study and will be asked for written informed consent to participate. From this point on, patients are either enrolled in the stepped-care program or will receive care as usual, depending on the general practice at which they are registered. An overview of the study design and patient flow is provided in Figure 1.

### Intervention

The intervention is modelled after the flexible stepped-care intervention developed by van ‘t Veer-Tazelaar et al. [26]. After receiving a specially developed training, practice nurses in the intervention practices will act as care managers and work together with the General Practitioner (GP) to provide the stepped-care treatment. The care managers coordinate the execution of the stepped-care program that consists of four evidence-based subsequent treatment steps, lasting 3 months each. The flow of participants in the intervention group through the stepped-care program depends on their depressive symptom level, measured using the PHQ-9 every 3 months during 1 year. Participants who still have elevated depressive symptom levels (i.e. a PHQ-9 score of 6 or more) after concluding a step, are offered participation in the next step. A score below the cut off point results in a period of watchful waiting until an elevated PHQ-9 score indicates the need for the subsequent step of the intervention. By only offering more intensive treatment to patients who continue to have elevated depressive symptom scores, it is expected that patients will receive treatment tailored to their needs and that available resources are more efficiently used by providing the more intensive treatments only to those who need this. Patients who meet the MINI diagnostic criteria for major depression at baseline or at 3, 6, 9 or 12 months are referred to their GP by the practice nurse for further assessment. The following treatment steps are offered to participants:

#### *Step 1: Watchful waiting*

The first 3 months consist of watchful waiting, because depressive symptoms often disappear spontaneously over time [26]. After inclusion and obtaining informed consent, patients are invited by their care manager for an introductory consultation. During this consultation patients will get acquainted with the care manager and they will receive an information brochure about mild depression with simple advices on how to cope with mild depressive symptoms. Patients will be informed about the stepped-care program and its rationale. In this step, no therapeutic intervention will take place.

#### *Step 2: Guided self-help treatment*

During this step, participants are offered a self-help course that is specially designed for patients with a chronic physical illness and depressive complaints [38]. During a visit to the general practice, the care manager will give the patient all necessary materials and explain the self-help course. Participants can work through the course at their convenience. In doing so, they will be supported by their care manager, who will contact them every other week by phone to monitor their progress. If it is clear that after two weeks the patient has not started the course yet, the care manager will use motivational interviewing techniques by phone to activate the patient. When this does not have the intended activating effect on the patient after two more weeks, the care manager invites the patient to come to the practice to discuss the current depressive symptoms (a PHQ-9 will be administered). When depressive symptoms still exist, patients are offered to progress early to step 3.

#### *Step 3: problem-solving treatment*

In this step, participants are offered Problem Solving Treatment (PST) by the care manager. PST is a brief cognitive behavioural intervention that focuses on practical skill building. It consists of a maximum of 7 sessions during which the stages of problem solving are explained and then applied to problems encountered in daily life. The goal of PST is to help patients regain control of their lives [39,40].

#### *Step 4: Referral to general practitioner*

Participants with continuously elevated PHQ-9 scores after 9 months will be referred to their general practitioner by the care manager for further assessment of their depressive symptoms. The GP receives a summary of the treatment provided to discuss with their patient.

#### Usual care

In the usual care practices, practice nurses and GPs will be blinded to which patients are participating in the study. They will not receive any training and will provide care as usual to all their patients according to existing clinical guidelines [41-43]. Participants in the usual care group will have unrestricted access to care as normally provided by their general practitioner. Their healthcare uptake (including use of prescribed medication) will be recorded.

### Training of the practice nurses

In the intervention practices, the practice nurses who are going to provide the stepped-care treatment will attend a two day training. This training focuses on how to implement the stepped-care program, how to provide guidance with the self help course using motivational interviewing techniques and how to provide the PST. The motivational interviewing techniques and the PST will be taught by a professional trainer. Before the trial starts, the trained practice nurses will perform two practice sessions of PST by telephone. These sessions will be audio taped and evaluated by the training staff to detect possible competence and/or adherence issues. During the trial, all practice nurses are regularly supervised by the training staff. Nurses can also contact the training staff to discuss any questions or problems, should they arise. When practice nurses have previously received training in motivational interviewing and/or PST, they are offered to follow a personalized training program.

### Outcomes

The primary clinical outcome is the cumulative incidence of DSM-IV major depressive disorder after 12 months according to the Mini International Neuropsychiatric Interview (MINI) [33,37]. The MINI will be administered by telephone at baseline and after 6 and 12 months after baseline by qualified research assistants.

Depression severity will be measured using the PHQ-9 [32,44]. The PHQ-9 is a widely used validated instrument to measure depression symptoms in general practice.

Quality of life will be measured using the EuroQol (EQ-5D) [45]). The obtained EQ-5D scores will be used to calculate utilities according to the Dutch tariff. QALYs will be calculated using the area-under-the-curve method with linear interpolation between time points.

For the economic evaluation, costs will be measured using the TiC-P questionnaire [46]. Costs that will be included are costs for healthcare utilization, informal care, and work absenteeism and presenteeism. Medication use will be retrieved from the patient’s pharmacy. If available, Dutch guideline prices will be used to value resource use. Medication use will be valued using prices of the Royal Dutch Society for Pharmacy. Lost productivity costs will be calculated according to the friction cost approach (friction period 154 days) using the mean age- and sex-specific income of the Dutch population. According to the friction cost approach a sick employee is replaced after a certain amount of time (the friction period) after which there are no lost productivity costs anymore. All costs will be adjusted to the year in which most data is collected using consumer price indices.

All secondary outcomes mentioned above will be administered at baseline, 3, 6, 9 and 12 months after inclusion through web-based questionnaires. If patients do not have access to the internet or prefer hard copies, we will provide these. In the intervention group, the PHQ-9 will also be administered by the nurse in the general practice for clinical monitoring and adjusting treatment when necessary.

Other secondary clinical outcomes include blood pressure, low-density lipoprotein (LDL) cholesterol and glycosylated haemoglobin (HbA1C). These outcomes will be measured at baseline and at 12 months follow-up and are performed as part of the usual care for this group of patients. Therefore, patients do not undergo any extra physical measurements.

To be able to control for possible confounders, demographics will be measured at baseline, along with personal and family history of mood disorders, using a subset of the Diagnostic Interview Schedule (DIS) [47]. The occurrence of co-morbid chronic illnesses will be assessed with the Dutch questionnaire chronic illnesses [48] at baseline and after 6 and 12 months, as well as locus of control [49]and social support [50]. All these measurements were previously used for the same purposes in the West Friesland Study [51]. In addition to this, anxiety symptoms will be measured at baseline, 3, 6, 9 and 12 months by the Hospital Anxiety and Depression Scale- Anxiety (HADS-A) [52].

After 12 months the uptake of the stepped-care program will be evaluated. The number of contacts with the care manager, the number of PST sessions, antidepressant use, and the number of referrals to the GP will be assessed by the care manager in the intervention group. Mental health care utilization outside the general practice will be assessed using the TiC-P questionnaire [46].

A process evaluation will be performed in which the barriers and facilitators of the stepped-care program and the experiences with the program will be evaluated. This will be done by organizing focus groups among volunteering patients, general practitioners and primary care nurses. In addition, satisfaction with the received care will be measured in all patients after 12 months by using the CSQ [53,54]. An overview of all measurements and instruments is provided in Table 1.

**Table 1:** Overview of all measurements and instruments

	**screening**	**baseline**	**3 months**	**6 months**	**9 months**	**12 months**
Depression symptoms (PHQ-2)	**x**					
Demographics		**x**				
Depression symptoms (PHQ-9)		**x**	**x**	**x**	**x**	**x**
Clinical depression (MINI)		**x**		**x**		**x**
Health care costs (Tic-P)		**x**	**x**	**x**	**x**	**x**
Quality of life (EQ-5D)		**x**	**x**	**x**	**x**	**x**
Anxiety (HADS-A)		**x**	**x**	**x**	**x**	**x**
Personal and family history (DIS)		**x**				
Social support		**x**		**x**		**x**
Existing chronic illnesses		**x**		**x**		**x**
Locus of control		**x**	**x**	**x**	**x**	**x**
Satisfaction with provided care (CSQ)						**x**

### Sample size

The trial is powered to detect a difference of 15% in the cumulative incidence rates of MINI/DSM-IV depressive disorder between the conditions after 1 year. The incidence rate is expected to be 30% in the usual care group and 15% in the intervention group based on findings from earlier studies [2,25,26]. Using a standard sample size calculation, 121 patients per group are needed, assuming a power of 0.8 and an alpha of 0.05. However, we need to correct for the fact that there is a multilevel setting with three levels: GPs, patients and measurements. Therefore we have first adjusted for the fact that we do not have one, but multiple measurements per patient. Assuming these measurements are clustered with an intraclass correlation (ICC) of 0.45, seventy-one patients per group are needed. Subsequently, we adjusted this figure further for clustering of patients within GP practices. Assuming an ICC of 0.05 for clustering of patients within the 30 GP practices, we need a total of 177 patients. Finally, we adjusted for a dropout rate of 25%, which means that 236 patients (118 patients per group) need to be included in this trial. This power calculation is based on the method described by Twisk in 2006 [55].

### Statistical analyses

Baseline data will be presented comparing the two treatment groups.

All analyses will be on an intention-to-treat basis. Differences between the stepped-care group and the usual care group will be tested using mixed model analyses. To test the cumulative incidence of depression over time, logistic mixed model analysis will be used. The obtained odds ratio describes the reduction in the risk of a MINI/DSM-IV depressive disorder in the intervention group relative to the control group. Linear and logistic mixed models (depending on the outcome) will also be used to test differences in symptoms of depression and anxiety and quality of life between both groups over time. If necessary, the models will be adjusted for confounders.

Sub group analyses will be performed to check for an interaction effect between depression severity at baseline and improvement in depressive symptoms at 12 months.

In case of unequal distributions of demographic variables between the two treatment groups, multivariate analyses techniques will be used to correct for these differences.

#### Economic evaluation

Both a cost-effectiveness analysis and a cost-utility analysis will be performed. Missing cost and effect data will be imputed using multiple imputation according to the MICE algorithm developed by Van Buuren [56]. The results of the imputed datasets will be pooled using Rubin’s rules [57]. Costs typically have a highly skewed distribution. Policy makers want to have information on the difference in mean total costs between the two treatment groups in order to be able to estimate the total health care budget needed for a specific condition [58]. Therefore, bias-corrected and accelerated bootstrapping with 5000 replications will be used to estimate 95% confidence intervals around the mean difference in total costs between the treatment groups. Incremental cost-effectiveness ratios (ICERs) will be calculated by dividing the difference in mean total costs between the treatment groups, by the difference in mean effects between the treatment groups. Bootstrapping will also be used to estimate the uncertainty surrounding the ICERs, which will be graphically presented on cost-effectiveness planes. Cost- effectiveness acceptability curves will be estimated as well. Cost-effectiveness acceptability curves show the probability that the stepped-care program is cost-effective in comparison with usual care for a range of different ceiling ratios (i.e. the willingness to pay for 1 extra recovered patient), thereby showing decision uncertainty [59].

## Discussion

Based on earlier studies, there is substantial evidence that stepped-care programs for depression among primary care patients are effective in both improving and preventing depression. However, evidence concerning the costs and effects of such programs among patients with DM2 and/or CHD and subthreshold depression in general practices is still missing.

Given the large number of DM2 and CHD patients with depressive symptoms, and the limited capacity for depression treatment in primary care, there is an urgent need to improve care for these patients. Since the presence of subthreshold depressive symptoms is the largest risk factor for developing major depression, targeting preventive treatments to patients at risk for developing a major depression is a viable option to reduce the burden of depression in primary care. Stepped-care interventions are a very promising method to achieve this goal. The key of stepped-care, delivered by a care manager, is that treatment is tailored to the needs and preferences of the patient while making the best use of available recourses. This will help optimizing health related outcomes, as well as keeping the treatment affordable by saving available resources for patients who really need them.

By testing a stepped-care program to prevent depression among high risk DM2 and/or CHD patients in a primary care setting, this study is highly clinically and societally relevant. Moreover, because a structured evaluation of the feasibility of the stepped-care program is explicitly taken into account in the study design, the results of this cluster randomised controlled trial will greatly contribute to the practical evidence about preventive treatment options for depression in patients with DM2 and/or CHD in general practice.

A strength of this study is that a pragmatic study design is employed; meaning that patients, treatments and procedures are similar to daily clinical practice. This greatly enhances the generalizability of the findings of the study and therefore the possibilities to implement the Step-Dep treatment in a real-life primary care setting.

The relatively short 12 month follow-up period may turn out to be a limitation of this trial. It is possible that the potential health benefits of participating in the stepped-care program will not yet be fully visible after one year. Especially the beneficial effects of having less depressive symptoms on health care utilisation, work absenteeism, and clinical outcomes such as Hb1Ac, blood pressure and LDL cholesterol, may take longer than the follow up period of one year to reach full development. Katon and his colleagues, for example, found a significant reduction in total health care costs among DM2 patients that participated in a stepped-care intervention to treat major depression after two years, but not yet after 12 months [30]. Other studies report similar results [60-62]. These findings can be explained by the fact that the extra costs of stepped-care interventions are made in the first year, while the cumulative effects of the treatment will become more and more visible after the treatment is finished. Therefore it might be necessary to perform a follow up study when the results indicate such a delay in cumulative effects.

Another possible limitation is that it is not possible to blind patients and caregivers to the randomisation due to the nature of the stepped-care program. However, considering the pragmatic design of the study, this is a true representation of clinical practice and this also enables us to measure all possible effects of the intervention. We try to counter possible contamination between treatments groups by using a cluster randomized controlled design, in which staff from usual care practices are not trained to perform the stepped-care program until after the follow up period. That way, caregivers cannot unintentionally apply aspects of the stepped-care program to their usual care for patients.

Overall, this study will provide valuable information about the cost-effectiveness and feasibility of a stepped-care program in primary care to prevent major depression in DM2/CHD patients with subthreshold depression, both from a clinical and societal perspective. When the stepped-care program proves to be cost-effective, the results of this study will offer a unique venture point for implementation of the stepped-care program into primary care and further research. The first results of this trial are expected in 2015.

## Abbreviations

CHD: Coronary Heart Disease; CSQ: Client Satisfaction Questionnaire; DIS: Diagnostic Interview Schedule; DM2: Type 2 Diabetes Mellitus; DSM-IV: Diagnostic and Statistical Manual of Mental Disorders-IV; EQ-5D: EuroQol- 5 dimensions; GPs: General Practices; HADS-A: Hospital Anxiety and Depression Scale-A; ICC: Intraclass correlation; ICERs: Incremental Cost-Effectiveness Ratios; ICPC: International Classification of Primary Care; LDL cholesterol: Low-Density Lipoprotein cholesterol; PHQ-2: Patient Health Questionnaire-2; PHQ-9: Patient Health Questionnaire- 9; PST: Problem Solving Treatment.

## Competing interests

The authors declare that they have no competing interests.

## Authors’ contributions

SvD constructed the design of the study and drafted the manuscript. AP constructed the design of the study and revised the manuscript. MCA, JB, PE and HvM developed the study, constructed the design and revised the manuscript. MvT participated in the design of the study and revised the manuscript. The final manuscript was read and approved by all authors.

## Pre-publication history

The pre-publication history for this paper can be accessed here:

http://www.biomedcentral.com/1471-244X/13/128/prepub

## Supplementary Material

Additional file 1 **ICPC codes used for recruitment.** This file provides a list of all registration codes used to identify potentially eligible patients in the medical electronic information system of participating general practices. Click here for file
