# Supplementary material for: Cost-effectiveness of a stepped-care intervention to prevent major depression in patients with type 2 diabetes mellitus and/or coronary heart disease and subthreshold depression: design of a cluster-randomized controlled trial
Source: BMC Psychiatry. 2013 May 7;13:128. doi: 10.1186/1471-244X-13-128 (PMC3654943; doi:10.1186/1471-244X-13-128)
Supplement: Additional file 1 — ICPC codes used for recruitment. This file provides a list of all registration codes used to identify potentially eligible patients in the medical electronic information system of participating general practices. [file 1471-244X-13-128-S1.pdf]

Additional file 1: ICPC codes used for recruitment

**ICPC codes**	**Omschrijving**
T90	Diabetes
T90.02	Diabetes Mellitus 2
K74	Angina pectoris
K74.01	Instabiele angina pectoris
K74.02	Stabiele angina pectoris
K75	Acuut myocardinfarct
K76	Andere/chronische ischemische hartziekte
K76.01	Coronairsclerose
K76.02	Vroeger myocardinfarct (> 4 wkn geleden)
